# Relationship between parental stress and attitude towards cochlear implantation outcomes in children in an Indian context

**DOI:** 10.1590/2317-1782/20212021125

**Published:** 2022-03-25

**Authors:** Saloni Piplani, Mohan Kumar Kalaiah, Usha Shastri

**Affiliations:** 1 Department of Audiology and Speech Language Pathology, Kasturba Medical College, Mangalore, Manipal Academy of Higher Education, Manipal, Karnataka, India; 2 Asha Kiran Speech and Language Habilitation Center, Jaipur, Rajasthan, India

**Keywords:** Parental Stress, Outcome of Cochlear Implant, Parental Attitude, Parental Stress Level, Cochlear Implant

## Abstract

**Purpose:**

The study was aimed to investigate the relationship between parental stress and attitude of parents towards the outcomes of cochlear implantation in an Indian scenario.

**Methods:**

A total of 59 parents of children with cochlear implantation participated in the study. The outcomes of cochlear implant was measured using Parental attitudes of various aspects of cochlear implantation questionnaire and parental stress was measured using parental stress scale. The questionnaires were circulated to participants and data was collected in the form of e-survey.

**Results:**

The present study showed that the parental stress level was similar among mothers and fathers. Further, the parental attitude towards communication abilities of children and education were positively correlated with the duration of cochlear implantation. Finally, a significant positive correlation was found between the parental stress and the parental attitude towards communication abilities of children and social skills.

**Conclusion:**

The present study showed a positive relationship between parental stress and parental attitude towards the outcomes of cochlear implantation for aspects of communication abilities and social skills.

## INTRODUCTION

Hearing is crucial for the development of speech and language abilities in children and communication. Hearing loss in the early childhood causes delay in the development of speech and language abilities which affects communication and academic achievement. The poorer communication abilities affect social skills and development of emotion and cognition. Cochlear implantation is a widely used treatment option for individuals with severe to profound hearing loss. It enables access to a broader range of speech sound frequencies and wider intensity range to individuals with severe to profound hearing loss. Several studies have compared the benefits of cochlear implant (CI) and hearing aids. The performance of CI users on tests of spoken language was found to be significantly better compared to hearing aid users^(1)^. In addition, the CI has a positive effect on the outcomes in terms of communication skills, functioning, well-being, self-reliance, social relations, and schooling^(2-6)^.

Parenting children with hearing loss is a challenge for parents^(7)^, thus they are at a higher risk for parental stress, impacting parent-child relationship and language learning outcomes. Several studies have investigated the levels of parental stress among parents of children with hearing loss and parents of children without hearing loss^(8-13)^. The above investigations have showed mixed findings. Few studies have reported higher stress level among parents of children with hearing loss^(8,9)^, no significant difference in stress level between groups^(11-13)^, and lower stress among parents of children with hearing loss^(10)^. But, studies measuring parental stress in the context of having a child with hearing loss have consistently reported higher stress among parents of children with hearing loss^(8,9)^. Further, studies comparing the parental stress level of parents of children with CI and parents of children using hearing aids have showed inconsistent findings^(7,13-15)^. Few studies reported lower stress among parents of children with CI^(7,14)^, higher stress among parents of children with CI^(15)^, and no difference in stress levels between the two groups^(13)^.

Several studies have investigated the relationship between parental stress and outcomes of children with CI^(9,11,16-19)^. In general, the findings of these investigations have showed a negative correlation between parental stress and outcomes of CI.

Faramarzi et al.^(19)^ investigated the relationship between parental stress and developmental skills of children with CI. Results showed a significant negative correlation between parental stress and language development (moderate), social development (weak), and communication development (moderate) of children. The regression analysis showed that 34% of variance in language development, 14% of variance in social development, and 29% of variance in communication development was explained via parental stress. These findings indicate that parental stress has a significant effect on developmental skills of children with CI. In Indian context, studies investigating the parental expectation from children with cochlear implants are available. However, studies investigating the relationship between parental stress and outcome of CI are rare. Thus, the present study was carried out to examine the relationship between parental stress and the attitudes of parents towards the outcomes of cochlear implantation in an Indian scenario.

## METHODS

The present study was a cross-sectional study. The study was approved by institutional ethics committee “Kasturba Medical College, Mangalore” (Protocol No: IECKMCMLR-10/2020/303) and the informed consent was obtained from each participant.

### Participants

A total of 59 parents of children with cochlear implantation participated in the study. Among them, 27 were mothers of children with CI and 32 were fathers of children with CI. Parents of children who had undergone cochlear implantation either in one ear or both ears were included for the study. The mean age of children with CI was 8.6 years (SD=4.2). Among the 59 children, 28 children were females (mean=7.8 years, SD=4.5) and 30 were males (mean=9.2 years, SD=3.8). Further, the mean age of children at diagnosis of HL, the mean age at CI, and the mean duration of use of CI was 1.5 years (SD=0.8), 3.4 years (SD=1.6), and 5.2 years (SD=3.7) respectively. The minimum age of children at diagnosis of HL, the minimum age at CI, and the minimum duration of use of CI was 4 months, 1.6 years, and 6 months respectively. All children used the CI regularly as reported by parents.

### Procedure

Parental stress scale^(20)^ was used to measure the level of stress experienced by parents of children who have undergone cochlear implantation. Parental attitudes of various aspects of cochlear implantation (PAVACI) questionnaire^(21)^ was used to assess the parent’s attitude towards the outcomes of CI. It measures the parental attitude towards the outcomes of cochlear implantation on aspects of communication skills, education skills, social skills, services provided by cochlear implantation centres, rehabilitation programs and decision-making process. The demographic characteristics of children such as age, gender, age of identification of hearing impairment, age of the children at the time of CI, and duration of CI were also collected. The data collection was carried out as an e-survey. The survey questionnaire was created using Google forms (Google Inc., Mountain View, CA, USA) and circulated to parents of children with CI by e-mail and through the social media platform WhatsApp.

### Data and statistical analysis

The level of stress experienced by parents was calculated as described by Bashiri et al.^(16)^. Total score was obtained by calculating sum score of responses to all questions. Reverse scoring was done for seven items (1, 2, 5, 6, 7, 17, and 18) of the parental stress scale. Similarly, the PAVACI was analysed as described in earlier investigation^(16)^. Total score was obtained for each domain of PAVACI, by finding sum of responses to all the items. Finally, the total score of parental stress scale and domains of PAVACI was converted to percentage. All statistical analysis was carried out using JASP software. Initially, the data was subjected to descriptive analysis to obtain mean scores, and to Shapiro-Wilk test to identify whether data is normally distributed. Independent samples ‘t’ test was used to investigate whether stress levels are significantly different between parents (mother and father). Mann-Whitney test was used to investigate whether outcome of CI among children was significantly different between gender (male and female). Pearson’s correlation analysis was used to investigate the relationship between parental stress and domains of PAVACI. Spearman’s correlation analysis was used to investigate the relationship between domains of PAVACI and duration of use of CI, and the age of children at implantation of CI.

## RESULTS

Responses of parents of children who had used the CI for less than one year and questionnaires with incomplete responses were excluded from further analysis. After excluding the data, responses of 57 parents of children with CI were available for further statistical analysis. Among them, 26 were mothers of children with CI and 31 were fathers of children with CI. Further, among the 57 children (mean=8.7 years, SD=4.2), 27 children were females (mean=7.9 years, SD=4.5) and 29 were males (mean=9.2 years, SD=3.8). Further, the mean age of children at diagnosis of HL, the mean age at CI, and the mean duration of use of CI was 1.5 years (SD=0.8), 3.3 years (SD=1.6), and 5.4 years (SD=3.7) respectively. The minimum age of children at diagnosis of HL, the minimum age at CI, and the minimum duration of use of CI was four months, 1.6 years, and one year respectively.

The mean stress level among parents of children with CI was 69.1 (SD=10.8). The mean stress level in fathers and mothers was 69.5 (SD=10.4) and 68.5 (SD=11.5) respectively. To investigate if the mean stress levels are significantly different between parents (father and mother), the data was subjected to further statistical analysis. The data was subjected to the Shapiro-Wilk test, and the result showed that the data was normally distributed [father (p=0.76); mother (p=0.825)]. Since the data was normally distributed, independent samples t-test was carried out with stress level as the dependent variable and parent as an independent variable. Results showed that the mean stress level was not significantly different between parents [t(55)=0.371, p=0.712].

Table 1 shows mean scores for domains of the parental attitude of various aspects of CI. The mean scores for domains communication, social skills, and education was higher in males compared to females. To investigate if the mean scores are significantly different between gender (male and female), the data was subjected to further statistical analysis. The Shapiro-Wilk test showed that scores of both males and females were normally distributed for domains communication [female (p=0.198); male (p=0.066)] and social skills [female (p=0.16); male (p=0.341)]. But, scores of males were not normally distributed for education [female (p=0.485); male (p=0.011)]. Since the scores of education were not normally distributed, it was subjected to the Mann-Whitney test with education as the dependent variable and gender as an independent variable. Scores of other domains were subjected to independent samples t-test with communication and social skill as the dependent variable and gender as an independent variable. Results showed that the mean scores were not significantly different between males and females for domain communication [t(54)=-1.279, p=0.206] and social skills [t(54)=-1.394, p=0.169]. But, gender had a significant effect on the mean scores of education [W=210.5, p=0.003].

**Table 1:** Mean scores and standard deviation (in parenthesis) for domains of the parental attitude of various aspects of cochlear implantation

**Domain**	**Female**	**Male**	**Total**
Communication	79.0 (7.9)	82.3 (10.6)	80.5 (9.6)
Education	73.8 (12.2)	83.1 (9.1)	78.4 (11.6)
Social skills	81.4 (10.1)	85.1 (9.9)	83.2 (10.1)
Services provided by CI centre			83.0 (8.1)
Rehabilitation program			78.9 (11.5)
Decision-making process			79.4 (8.7)
Overall			81.3 (6.7)

The relationship between parental stress and attitudes towards various aspects of CI was investigated by measuring the correlation between stress level and scores on domains of PAVACI. Results of correlation analysis are shown in Table 2. It showed a significant positive correlation between parental stress and communication abilities and parental stress and social skills. In addition, a positive correlation was also found between parental stress and education and parental stress and services provided by the CI centre, but the correlation was not significant. Further, the relation of parental stress with the age of children at diagnosis, age of children during CI, and duration of use of CI was investigated using Spearman’s correlation analysis. Results showed no significant correlation between parental stress and age of children at diagnosis (rho=0.09, p=0.509), parental stress and age of children at CI (rho=-0.111, p=0.417), and parental stress and duration of use of CI (rho=0.042, p=0.758). In addition, the relation between scores of PAVACI with the age of children during CI and duration of use of CI was investigated using Spearman’s correlation analysis. The findings of correlation analysis are shown in Table 3. Results showed a significant positive correlation between duration of use of CI and communication and parental stress and duration of use of CI and education.

**Table 2:** Findings of correlation analysis between parental stress and domains of parental attitudes towards various aspects of cochlear implantation questionnaire

**Domain**	**Pearson’s r p-value**	**Spearman’s rho p-value**
Communication	0.353** 0.007	
Education		0.186 0.166
Social skills	0.437*** < 0.001	
Services provided by CI centre	0.201 0.133	
Rehabilitation program	0.114 0.400	
Decision-making process	-0.062 0.646	

** p<0.01;

*** p<0.001

**Table 3:** Findings of Spearman’s correlation analysis between the domains of parental attitudes towards various aspects of cochlear implantation and age of children during cochlear implantation and duration of use of cochlear implant

**Domain**		**Implantation**	**Usage**
Communication	Spearman’s rho p-value	-0.146 0.284	0.274* 0.041
Education	Spearman’s rho p-value	0.149 0.273	0.293* 0.028
Social skills	Spearman’s rho p-value	-0.119 0.384	0.094 0.489

* p<0.05;

## DISCUSSION

The present study aimed to investigate the relationship between parental stress and their attitudes towards outcomes of CI in an Indian scenario. The findings of the present study showed that the mean stress level among parents of children with CI was 69.05. In comparison to the results of earlier investigation^(16)^, the stress levels among Indian parents was higher. Further, the present study showed that the stress level in mothers and fathers were similar. This finding in the present study is consistent with findings of earlier investigations^(16,22)^. In contrast, higher stress and anxiety has been reported among mothers of children with CI^(23)^. The higher levels of stress among mothers has been attributed to the higher anxiety levels which is common among mothers. Several studies in literature have reported higher stress levels among parents of children with hearing loss, thus findings of the present study is consistent with results of earlier investigations. Higher stress among parents of children with hearing loss has been attributed to several factors. Quittner et al.^(9)^ attributed higher stress among parents of children with hearing loss to behavioural problems, language delays, and reduced parent-child interaction due to hearing loss. Abbas et al.^(24)^ attributed higher stress to communication gap, child’s future, lack of income to fulfil the child’s needs, and inability to express feelings. In addition, factors such as age of diagnosis, degree of hearing loss, language abilities of child, mode of communication, unrealistic communication and social expectations, participation restriction in everyday activities, social skills, family income, parental support, cost of CI, and damage to internal device are causal factors of stress^(7,10,11,13,16,17,25,26)^.

The present study found a significant positive correlation between the duration of CI and the parental attitude towards the outcomes of CI for aspects of communication abilities and education. A similar finding was reported by earlier investigation^(16)^. In addition, several studies have reported a significant positive relationship between parental attitude towards outcomes of CI with categorization of auditory performance abilities and speech intelligibility rating which are formal tests to measure outcome of CI^(16,27)^. The above findings suggest that communication abilities and education skills of children with CI improved with increase in the duration of cochlear implantation. Studies measuring the long term effects of CI have reported an increase in speech perception, language outcomes, use of oral language, and ability to function in a mainstream environment with usage of CI in children^(2-6)^.

Finally, the present study showed a positive relationship between parental stress and parental attitude towards the outcomes of CI for aspects of communication abilities and social skills. This finding was not expected as the majority of the investigations have reported a negative relationship between parental stress and the CI outcomes^(9,11,16-19,26)^. Further, Davies^(18)^ reported no correlation between parental stress and parental attitude towards communication skills of children with hearing loss. The different results found in this study in relation to the literature could be due to a limitation of the present study where child’s language and auditory skills were not analysed. Further, the present study showed no correlation between parental stress and parental attitude towards the outcomes of CI for aspects of rehabilitation program and decision-making process. These findings are consistent with results of Bashiri et al.^(16)^. The positive relationship between parental stress and outcomes from the CI observed in the present study could be due to high expectation levels of parents, insecure attachment of parents with their children, and poor accuracy of questionnaire-based investigations. Kumar et al.^(28)^ investigated the relationship between parental stress and outcomes of children with CI among Indian parents. Their results showed high expectations towards child’s communication, social participation, and educational skills. Thus, high expectation of parents could be one of the factors for the observed findings in the present study. Similarly, Pipp-Siegel et al.^(10)^ assessed parental stress, coping and attachment in families. It showed that the parental stress was significantly associated with insecure attachments of parents. John and Robins^(29)^ reported the accuracy and bias in self-perception and the individual differences in self enhancement and self-diminishment. They found poor accuracy among self-perception questionnaires which can be subjected to bias.

## CONCLUSION

Findings of the present study showed higher stress among parents of children with CI. These findings emphasize measuring parental stress level among parents of children with CI to assist them when required. The present study helps us to understand the importance of parental counselling to reduce the stress levels among parents of children with CI.
